# Change in Electrical/Mechanical Properties of Plasma Polymerized Low Dielectric Constant Films after Etching in CF_4_/O_2_ Plasma for Semiconductor Multilevel Interconnects

**DOI:** 10.3390/ma16134663

**Published:** 2023-06-28

**Authors:** Namwuk Baek, Yoonsoo Park, Hyuna Lim, Jihwan Cha, Taesoon Jang, Shinwon Kang, Seonhee Jang, Donggeun Jung

**Affiliations:** 1 Department of Physics, Sungkyunkwan University, Suwon 16419, Republic of Korea; 2 Foundry Metal Technology Team, Samsung Electronics, 190 Seoku-dong, Hwaseong-si 18448, Republic of Korea; 3 Department of Mechanical Engineering, University of Louisiana at Lafayette, Lafayette, LA 70503, USA

**Keywords:** low dielectric material, intermetal dielectric, tetrakis(trimethylsilyoxy)silane, reactive ion etching, plasma-enhanced chemical-vapor deposition

## Abstract

As semiconductor chips have been integrated to enhance their performance, a low-dielectric-constant material, SiCOH, with a relative dielectric constant *k* ≤ 3.5 has been widely used as an intermetal dielectric (IMD) material in multilevel interconnects to reduce the resistance-capacitance delay. Plasma-polymerized tetrakis(trimethylsilyoxy)silane (ppTTMSS) films were created using capacitively coupled plasma-enhanced chemical vapor deposition with deposition plasma powers ranging from 20 to 60 W and then etched in CF_4_/O_2_ plasma using reactive ion etching. No significant changes were observed in the Fourier-transform infrared spectroscopy (FTIR) spectra of the ppTTMSS films after etching. The refractive index and dielectric constant were also maintained. As the deposition plasma power increased, the hardness and elastic modulus increased with increasing ppTTMSS film density. The X-ray photoelectron spectroscopy (XPS) spectra analysis showed that the oxygen concentration increased but the carbon concentration decreased after etching owing to the reaction between the plasma and film surface. With an increase in the deposition plasma power, the hardness and elastic modulus increased from 1.06 to 8.56 GPa and from 6.16 to 52.45 GPa. This result satisfies the hardness and elastic modulus exceeding 0.7 and 5.0 GPa, which are required for the chemical–mechanical polishing process in semiconductor multilevel interconnects. Furthermore, all leakage-current densities of the as-deposited and etched ppTTMSS films were measured below 10^−6^ A/cm^2^ at 1 MV/cm, which is generally acceptable for IMD materials.

## 1. Introduction

As semiconductor chips have become more integrated, their pitch size has been reduced to improve their performance. This reduction in pitch size can decrease the gate delay in field-effect transistors [1,2]. However, the reduced pitch size causes an increase in the resistance-capacitance delay (RC delay), which results in the degradation of the performance of semiconductor chips. To resolve this problem, aluminum (Al) has been replaced by copper (Cu) because the resistivity of Cu (1.67 μΩ⸱cm) is smaller than that of Al (2.7 μΩ⸱cm). Traditional interlayer dielectric silicon oxide (SiO_2_) has been replaced by low dielectric constant (low-*k*) materials with a relative dielectric constant below 3.9 in multilevel interconnects [3,4]. As a low-*k* material, SiCOH films have been widely employed by plasma-enhanced chemical vapor deposition (PECVD) of precursors composed of Si-O and hydrocarbon functional groups [5,6,7,8,9]. In our previous work, tetrakis(trimethylsilyoxy)silane (TTMSS, C_12_H_36_O_4_Si_5_) was introduced as the precursor to fabricate SiCOH films [10,11,12,13]. It was reported that the SiCOH films showed low-*k* values in the range of 2.1 to 3.57 with good mechanical properties ranging from 7.12 to 41.4 GPa [11]. SiCOH films are referred to as plasma-polymerized tetrakis(trimethylsilyoxy)silane (ppTTMSS) films because plasma sources are used to generate a gas discharge that provides energy to activate or fragment molecular precursors and deposit polymer thin films. On the other hand, Cu dry etching is extremely challenging because it is difficult to remove byproducts (chlorides or fluorides) that are nonvolatile and have high boiling points above 1000 °C. Thus, the interconnect integration changed from metal (Cu) patterning followed by dielectric filling to dielectric patterning followed by metal filling with Cu. This is the so-called “damascene” process, which has raised the demand for low-*k* patterning by plasma etching. Unfortunately, the damascene process requires plasma exposure for etching, which can damage low-*k* films [14]. As a result, low-*k* films exhibit increased *k* values and leakage-current densities after plasma exposure [15]. In this study, the following investigations were performed to evaluate the suitability of ppTTMSS films as intermetal dielectric (IMD) materials, even after plasma exposure for etching. The effect of reactive ion etching (RIE) on ppTTMSS films was investigated to understand the changes in the chemical properties of the etched film surface. The RIE process was performed using plasma with CF_4_ and O_2_ gases. The etch rate was related to the chemical structures of the ppTTMSS films depending on the deposition plasma power. The refractive index, electrical properties including *k* value and leakage-current density, and mechanical properties including hardness and elastic modulus were also studied to evaluate the suitability of the IMD materials [16,17].

## 2. Materials and Methods

The ppTTMSS films were deposited using a capacitively-coupled plasma-enhanced chemical vapor deposition (PEVCD) system with a 13.56 MHz radio frequency (RF) power supply. A TTMSS precursor (Sigma Aldrich, St. Louis, MO, USA, 99% purity) was used. TTMSS has a cross-shaped structure composed of four oxygen atoms attached to a central silicon atom, and each oxygen atom is bonded to another silicon atom linked with three CH_3_ molecules. Two types of 4-inch Si wafers were used as substrates: phosphorus-doped n-type Si (100) with a resistivity of 1–10 Ω·cm and highly boron-doped p++-type Si (100) with a resistivity of less than 0.005 Ω·cm. They were then cleaned by ultrasonication in acetone and ethanol for 5 min each. To vaporize the molecules from the precursors, a bubbler containing the TTMSS precursor was heated to 95 °C, and an argon (Ar) gas flow rate of 60 sccm with a purity of 99.999% was introduced into the bubbler as a carrier gas, which transported the vaporized precursors to the process chamber. The ppTTMSS films were deposited at 25 °C and a working pressure of 80 Pa. Under the same conditions, the chamber pressure, when only Ar carrier gas was supplied to the chamber at a flow rate of 60 sccm, was 50 Pa. The deposition plasma power was varied in the range of 20 to 60 W. 

**Figure 1:**
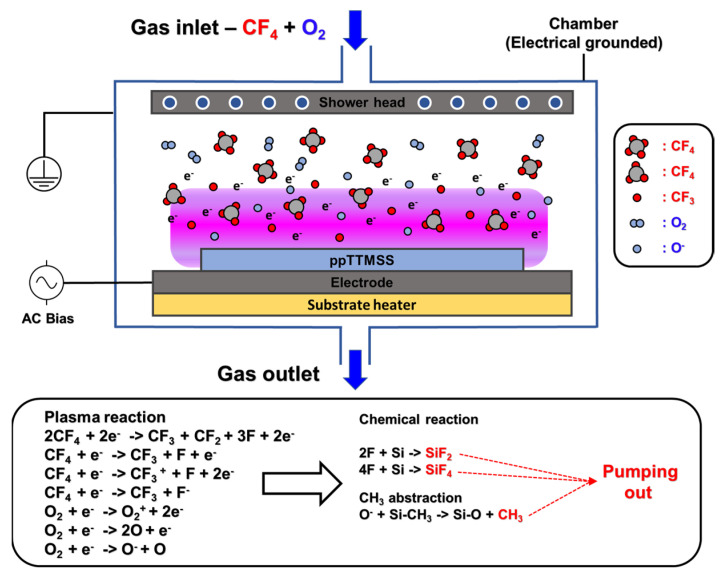
Schematic diagram of the etch process of the ppTTMSS film using CF_4_ + O_2_ gas plasma.

Figure 1 illustrates the RIE process of ppTTMSS film. The carbon tetrafluoride/oxygen (CF_4_/O_2_) gas injected into the chamber is activated by plasma to create radicals such as O and F and the active species react with the surface of the thin film to cause etching of the ppTTMSS film surface. Both the plasma reaction and chemical reaction in the chamber occur as indicated at the bottom of the figure. After the reaction, byproducts, such as SiF_x_ and CH_x_, could be evacuated from the chamber through the pump. The ppTTMSS films were etched for 5 min using the RIE process with carbon tetrafluoride/oxygen (CF_4_/O_2_) gas chemistry at gas flow rates of 5/5 sccm. The pressure in the RIE process chamber was 96 Pa. A capacitance-coupled plasma system was used for the RIE at 13.56 MHz. The bias power was maintained at 20 W.

The thickness and refractive index (n) of the ppTTMSS films were measured using an ellipsometer (M-2000, J.A. Woolam Co., Lincoln, NE, USA). The thicknesses of the ppTTMSS films were confirmed by field-emission scanning electron microscopy (FESEM; JSM-7600F, Jeol, Tokyo, Japan). To investigate the chemical composition of the ppTTMSS films, X-ray photoelectron spectroscopy (XPS; VG Microtech, ESCA2000, London, UK) was employed with a twin anode (13 kV) of Al-Kα (1486.6 eV) and Ma-Kα (1253.6 eV). The beam and filament currents of the X-ray source were 15 mA and 4.5 A, respectively, and the scanning step was 0.1 eV. Owing to the high beam energy, XPS measurements were conducted at a depth of ~10 nm from the surface without Ar sputtering. Fourier transform infrared (FTIR) spectroscopy (Bruker, VERTEX7, Billerica, MA, USA) was used to determine the chemical structures of the ppTTMSS films. FTIR absorption spectra were scanned 64 times with wavenumbers ranging from 4000 to 600 cm^−1^ with a resolution of 4 cm^−1^. The peaks obtained from the FTIR and XPS spectra were deconvoluted into their constituent peaks using Gaussian peak fitting with OriginPro software. The analysis of chemical and optical properties mentioned above used n-type Si-wafer samples. To investigate the electrical properties of the ppTTMSS films, a metal/insulator/metallic silicon wafer (MIS) structure consisting of Al/ppTTMSS/p++-Si was used. To measure the *k* value of the ppTTMSS films, an inductance, capacitance, and resistance (LCR) meter (4287 A, Agilent, Santa Clara, CA, USA) was employed at an alternating current power source with a frequency of 1 MHz and voltage of 100 mV. The leakage-current densities of ppTTMSS films were measured using an electrometer (6617 B, Keithley, Cleveland, OH, USA). The hardness and elastic modulus were measured by a load- and depth-sensing indentation technique with a nanoindenter (NanoTest Vantage Platform, Micro Materials, Anaheim, CA, USA) using an n-type Si wafer sample. The nanoindenter was measured at a depth of 30 to 40% of the film thickness to avoid the influence of surface oxide film and Si wafer.

## 3. Results and Discussion

**Figure 2:**
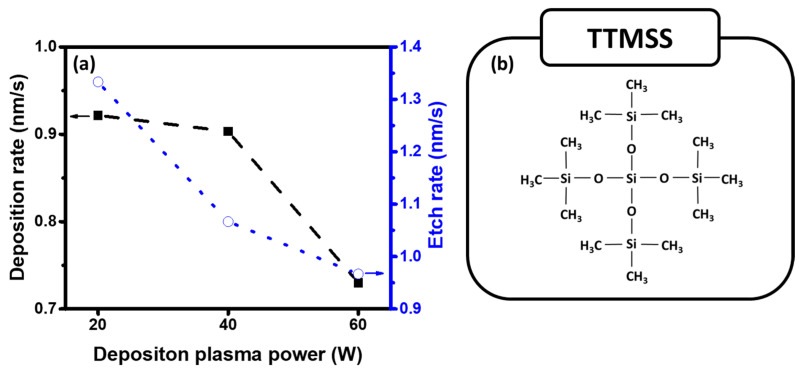
(**a**) Deposition and etch rates of the ppTTMSS films as a function of the deposition plasma power and (**b**) Structural diagram of ‘TTMSS’ precursor.

Figure 2 shows the deposition and etch rates of the ppTTMSS films as a function of the deposition plasma power. As the deposition plasma power increased from 20 to 60 W, the as-deposited samples were fabricated at 553, 542, and 438 nm, and the deposition rate decreased from 0.92 to 0.73 nm/s. After the RIE process, the thickness of the ppTTMSS films decreased to 513, 510, and 409 nm, and the etching rate decreased from 1.33 to 0.96 nm/s. An increase in the deposition plasma power can cause an increase in the density of ppTTMSS films by forming a SiO_2_-like structure [10,11]. The SiO_2_-like ppTTMSS film is developed by the competition between ablation and polymerization (CAP) mechanisms during the process of plasma polymerization [18,19]. Two processes occur simultaneously: ablation, which removes surface molecules, and polymerization, which deposits the surface monomer. These two processes are in competition, and the deposition rate can increase or decrease depending on whether the polymerization process or the ablation process is dominant. It was also found that the polymer deposition hardly occurred at a very low flow rate and the deposition rate decreased with increasing deposition plasma power [18,19]. Two major factors that influence the balance between polymer formation and ablation are considered to be the control of ablation due to reactive species by chemical reactions and plasma conditions, particularly the plasma energy density [18,19]. In this study, as the deposition plasma power increased from 20 W to 60 W, the deposition rate decreased. Thus, it is likely that the decreased deposition rate was caused by either a low flow rate or ablation in the plasma. However, the deposition plasma power could affect the changes in the chemical-bonding configurations of the films during deposition. The dissociation energy of chemical bonds depends on the bond type: C-H (3.5 eV), Si-C (4.7 eV), and Si-O (8.3 eV) [14,20]. More hydrocarbon-related molecules induced by C-H and Si-C were considered to appear in the films. However, as they increased, a large portion of the molecular precursors decomposed into small fragments of Si, O, and C, leading to SiO_2_-like films. It is possible that more SiO_2_-like structures, rather than carbon-related structures, induced lower etch rates at a higher deposition plasma power. 

**Figure 3:**
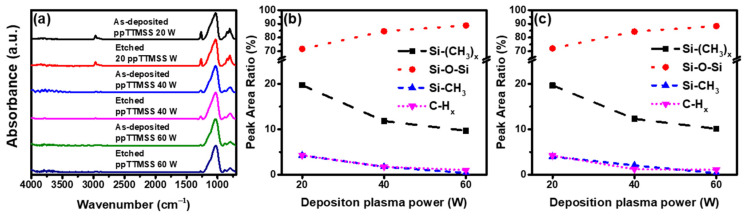
(**a**) FTIR absorption spectra of as-deposited and etched ppTTMSS films and peak-area ratios of Si-O-Si, Si-(CH_3_)x, Si-CH_3_, and C-Hx in the FTIR absorption spectra for (**b**) as-deposited and (**c**) etched ppTTMSS films.

Figure 3a shows the FTIR absorption spectra of the as-deposited and etched ppTTMSS films as a function of the deposition plasma power in the range of 4000 to 400 cm^−1^. The absorption band in the range of 3100 to 2800 cm^−1^ corresponds to the C-Hx (x = 2, 3) stretching vibration mode. The peak at 1300 to 1260 cm^−1^ originated from the Si-CH_3_ bond. The absorption band centered at 1260 to 950 cm^−1^ was assigned to the Si-O-Si stretching vibration mode. The absorption band at 900 to 750 cm^−1^ corresponded to the Si-(CH_3_)x (x = 1, 2, 3) bending vibration [21,22,23,24]. The Si-O-Si stretching vibration mode had the highest intensity among these peaks because the ppTTMSS films were mostly composed of siloxane induced from the molecular structure of the TTMSS precursor. As the deposition plasma power increased, the Si-O-Si stretching mode became dominant, and the intensities of the three hydrocarbon-related peaks diminished. There were few changes in these peaks after the etching of the ppTTMSS films and their peak-area ratios were analyzed for comparison. Figure 3b,c represent the peak-area ratios of the Si-O-Si and hydrocarbon-related peaks of the as-deposited and etched ppTTMSS films, respectively. For as-deposited ppTTMSS films, the peak-area ratio of Si-O-Si increased from 71.76 to 88.83%, and those of hydrocarbon-related peaks, including Si-(CH_3_)x (x = 1, 2, 3), Si-CH_3_ and C-Hx (x = 2, 3), decreased with an increase in the deposition plasma power from 20 to 60 W. Furthermore, the peak-area ratios of Si-(CH_3_)x, Si-CH_3_, and C-Hx decreased from 19.68 to 9.69%, from 4.28 to 0.42%, and from 4.28 to 1.06%, respectively. For etched ppTTMSS films, with an increase in deposition plasma power, trends in peak-area ratios, similar to those of the as-deposited films, were observed. As the deposition plasma power increased from 20 to 60 W, the peak-area ratio of Si-O-Si increased from 71.11 to 88.40% and those of Si-(CH_3_), Si-CH_3_, and C-Hx decreased from 19.65 to 10.11%, from 4.02 to 0.37%, and from 4.22 to 1.12%, respectively. 

**Figure 4:**
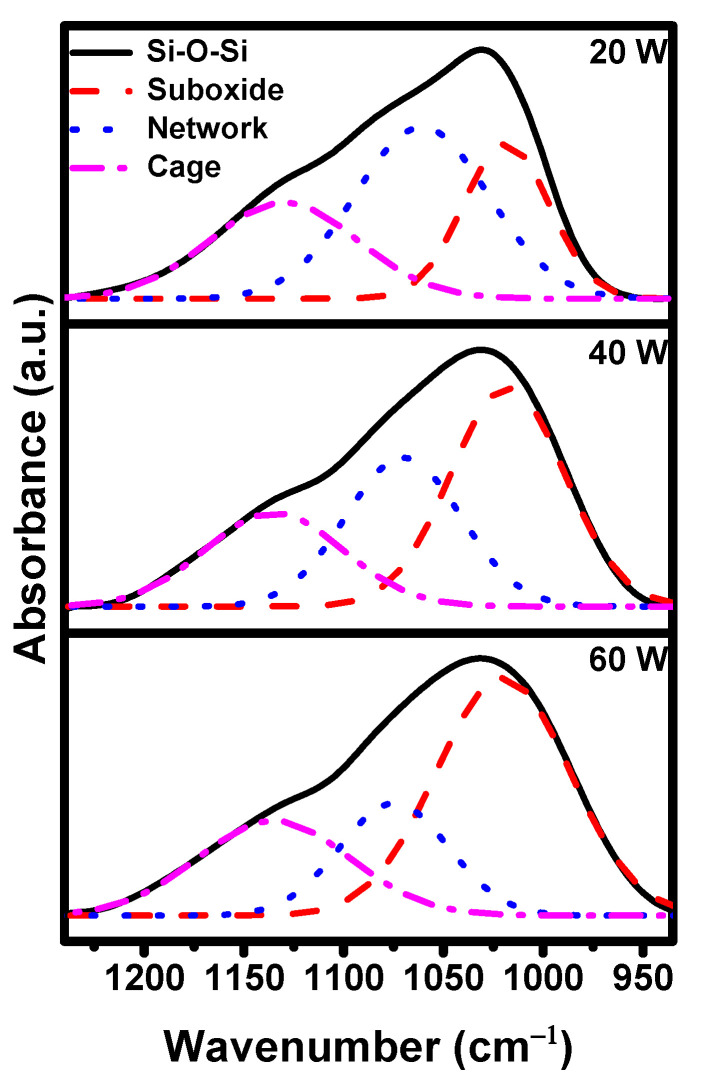
Deconvoluted absorption spectrum of as-deposited ppTTMSS films for suboxide, network, and cage structures, depending on the deposition plasma power.

Figure 4 presents the deconvolution of dominant Si-O-Si peaks of the as-deposited ppTTMSS films for deposition plasma powers ranging from 20 to 60 W. Similar deconvoluted peaks of etched ppTTMSS films were obtained. Three deconvoluted Si-O-Si peaks, including suboxide, network, and cage structures, were defined according to the Si-O-Si bonding angle [21,25]. The peak located at approximately 1030 cm^−1^ was allocated to the vibration of the suboxide structure with a bonding angle lower than 144° [26,27,28]. The peak at 1070 cm^−1^ was assigned to the vibration of the network structure with a bonding angle of ~144° [26,28]. The peak at 1140 cm^−1^ was ascribed to the vibration of the cage structure with a bonding angle larger than 144° [29]. As the deposition plasma power increased, the suboxide structure became dominant, whereas the network structure became more recessive. The peak-area ratios of the deconvoluted Si-O-Si peaks for both the as-deposited and etched ppTTMSS films are shown in Table 1. As the deposition plasma power increased from 20 to 60 W, the peak-area ratios of suboxide structure for the as-deposited ppTTMSS films increased from 27 to 56%, whereas those of the network and cage structures decreased from 45 to 22% and from 28 to 22%, respectively. The peak-area ratios remained almost the same for the etched ppTTMSS films.

**Figure 5:**
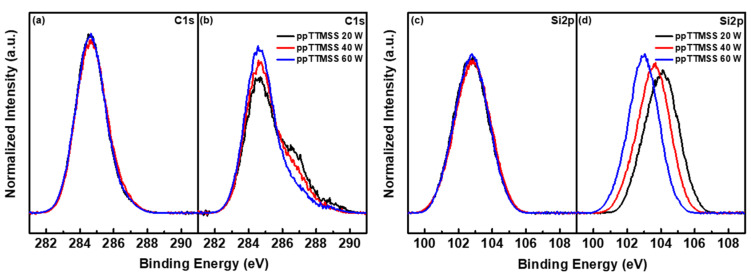
C1s peaks of (**a**) as-deposited and (**b**) etched ppTTMSS films and Si2p peaks of (**c**) as-deposited and (**d**) etched ppTTMSS films in XPS spectra.

The XPS spectra of the ppTTMSS films were studied to investigate the effect of CF_4_/O_2_ etching on their surface chemistry. Figure 5a,b are plotted as the normalized C1s peaks of the as-deposited and etched ppTTMSS films, respectively. Figure 5c,d show the normalized Si2p peaks of the as-deposited and etched ppTTMSS films, respectively. There were little changes in the C1s and Si2p peaks of the as-deposited films when the deposition plasma power increased from 20 to 60 W. For etched ppTTMSS films, a shoulder peak at approximately 287 eV was observed in the C1s peaks, and chemical shifts to a higher binding energy occurred with 104.03, 103.57, and 102.97 eV for 20, 40, and 60 W in the Si2p peaks of etched ppTTMSS films, respectively. The chemical shifts of the Si2p peaks could be caused by an increased link with oxygen and fluorine because oxygen and fluorine have more electron affinity than carbon and silicon [30]. The atomic concentrations and peak-area ratios of the deconvoluted C1s peaks of the as-deposited and etched ppTTMSS films are presented in Table 2. Consistent with the XPS spectra of the as-deposited ppTTMSS films, there were little changes in the atomic concentrations for s ranging from 20 to 60 W. When the deposition plasma power increased from 20 to 60 W for as-deposited ppTTMSS films, the concentration of oxygen increased from 28.37 to 30.96%, whereas the concentrations of silicon and carbon decreased from 28.11 to 26.56% and 43.52 to 42.48%, respectively. For etched ppTTMSS films, however, a relatively higher concentration of oxygen was measured, which decreased from 53.67 to 42.24% as the deposition plasma power increased from 20 to 60 W. In comparison with the as-deposited ppTTMSS films, a dramatic reduction in carbon concentration was observed, which increased from 14.92 to 31.00% as the deposition plasma power increased from 20 to 60 W. The concentration of silicon did not change much compared to those of carbon and oxygen after the RIE process, decreasing from 29.17 to 24.74% as the deposition plasma power increased from 20 to 60 W. Additionally, the concentration of fluorine was measured in the range of 1.99 to 2.24% owing to the CF_4_/O_2_ plasma etching. The decrease in carbon concentration and increase in oxygen concentration after the RIE process can be explained by the reaction between the CF_4_/O_2_ plasma and the film surface. For etching ppTTMSS films using CF_4_ plasma, it is desirable to form F-radicals through electron impacts in the plasma and volatile Si compounds such as SiFx. This requires breaking the Si-O, Si-C, and C-F bonds and the formation of Si-F bonds. By adding oxygen to the plasma, carbon can be removed via the formation of CO and CO_2_ gases, which are easily pumped away. This decreases the amount of carbon available to form CFx radicals, increasing the relative F concentration in the plasma and the etch rate. Meanwhile, oxygen can create SiO_2_ on the surface, resulting in slower etching in CF_4_ chemistry. The etch rate is not strictly proportional to the concentration of F radicals because of the competition between F atoms and O atoms for the active Si surface site.

**Figure 6:**
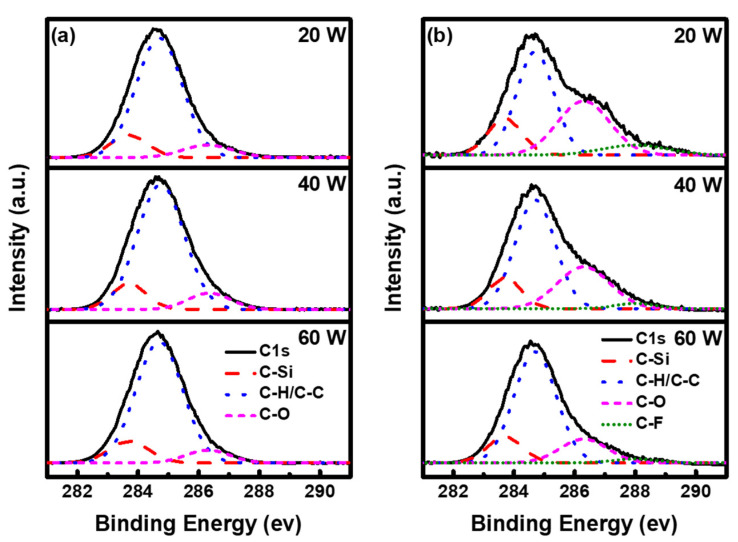
Deconvoluted C1s peak of (**a**) as-deposited and (**b**) etched ppTTMSS films for C-Si, C-H/C-C, C-O, and C-F subpeaks.

Figure 6a,b show the deconvoluted C1s peaks of the as-deposited and etched ppTTMSS films, respectively. Table 3 shows the peak-area ratios of the deconvoluted C1s peaks of the as-deposited and etched ppTTMSS films. Subpeaks C-Si, C-C/C-H, and C-O were defined by their associated binding energies of 283.8, 284.8, and 286.3 eV for as-deposited ppTTMSS films, and additional C-CF was observed at a binding energy of 287.0 eV for etched ppTTMSS films [31]. The subpeak of C-H/C-C was predominant with peak-area ratios of 79 to 80%, followed by 12% of C-Si and 8–9% of C-O for as-deposited films. After CF_4_/O_2_ plasma etching, there was little change within the range of 12 to 14% in the peak-area ratios of the C-Si peak. However, the peak-area ratios of the oxygen-related C-O peak increased, ranging from 16 to 33%, which is consistent with the increase in oxygen concentration after CF_4_/O_2_ plasma etching. The concentration of oxygen decreased from 53.67 to 42.24%, and the peak-area ratio of C-O decreased from 33 to 16% with an increase in the deposition plasma power from 20 to 60 W. The peak-area ratios of the carbon-related C-C/C-H peaks decreased, ranging from 45 to 69%, which is in accordance with the decrease in carbon concentration after CF_4_/O_2_ plasma etching. The concentration of carbon increased from 14.92 to 31.00%, and the peak-area ratio of C-C/C-H increased from 45 to 69% with an increase in the deposition plasma power from 20 to 60 W. The C-CF peak also appeared owing to CF_4_ chemistry and its peak-area ratios decreased from 8 to 2% as the deposition plasma power increased from 20 to 60 W.

**Figure 7:**
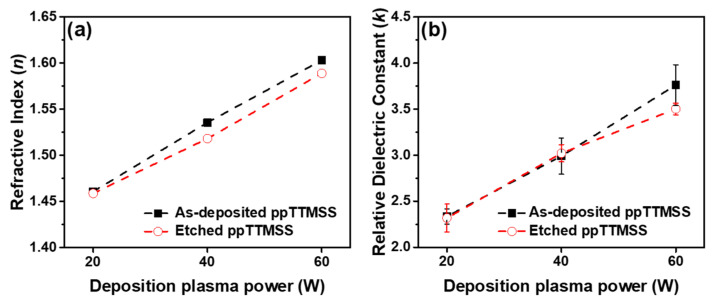
(**a**) Refractive index and (**b**) relative dielectric constant of ppTTMSS films as a function of deposition plasma power.

Figure 7a,b present the refractive index (*n*) and relative dielectric constant (*k*) of the as-deposited and etched ppTTMSS films, respectively. The *n* values of as-deposited ppTTMSS films increased from 1.46 to 1.60 with an increase in the deposition plasma power from 20 to 60 W. After etching the ppTTMSS films, the *n* values were slightly lower than those of the as-deposited films, increasing from 1.45 to 1.58 when the deposition plasma power increased from 20 to 60 W. It has been reported that the *n* value is closely related to the density of the films [32,33]. It is also known that the refractive index is affected by the carbon content of the film; an increase in the refractive index could result from a decrease in the carbon content. This is consistent with the fact that carbon concentration decreased from 43.52 to 42.48% when the deposition plasma power increased from 20 to 60 W. It was noted that the refractive index did not change significantly for etched ppTTMSS films, although the carbon content decreased from 14.92 to 31.00%. This could be caused by the change in the surface chemistry resulting from the increased oxygen and fluorine content. The *k* values of the as-deposited ppTTMSS films increased from 2.33 to 3.76 and those of etched ppTTMSS films increased from 2.31 to 3.65 as the deposition plasma power increased from 20 to 60 W. Pore formation is known to be derived from the cage structure in ppTTMSS films and contributes to the reduction in *k* values [21]. The peak-area ratio of cage structures, which could be related to pores inside the ppTTMSS film, reduced from 28 to 22% for both as-deposited and etched ppTTMSS films as the deposition plasma power increased from 20 to 60 W. This behavior can be explained by an increase in the fraction of cage structures, resulting in decreased *k* values [21]. It is generally accepted that the refractive index is directly proportional to the *k* value [34,35,36]. In accordance with this behavior, both the refractive index and the *k* value increased as the deposition plasma power increased. The *k* values remained almost the same after the ppTTMSS films were etched. This explains why etching did not significantly affect the electrical properties, which is beneficial for the stability of the ppTTMSS films. It was assumed that the etching process mostly affected the surface chemistry without causing any significant damage to the film.

**Figure 8:**
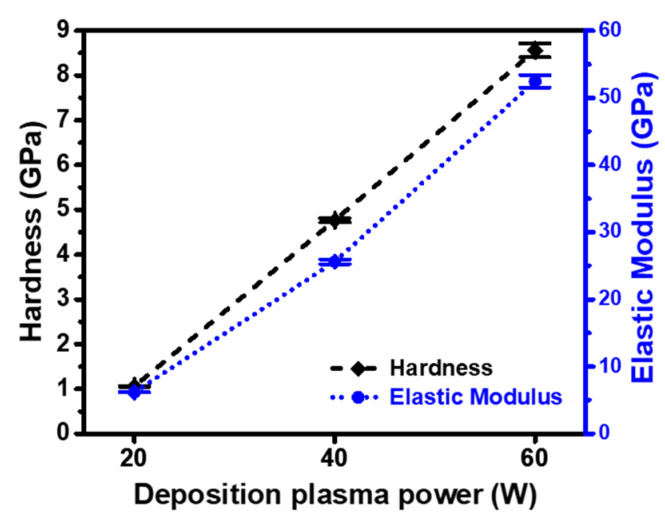
Hardness and elastic modulus of as-deposited ppTTMSS films as a function of deposition plasma power.

To evaluate whether the ppTTMSS films are suitable as IMD materials, their mechanical properties, including hardness and elastic modulus, were analyzed, as shown in Figure 8. Increased density has been shown to enhance mechanical strength [37]. With an increase in the deposition plasma power, the hardness and elastic modulus increased from 1.06 to 8.56 GPa and from 6.16 to 52.45 GPa, respectively. For a higher deposition plasma power of 60 W, more SiO_2_-like structures were observed, leading to a higher density and mechanical strength. Enhanced mechanical properties could lead to a smaller etch rate because films with high mechanical strength are resistant to physical etching due to ion collisions. It has been reported that the hardness and elastic modulus exceeding 0.7 and 5.0 GPa, respectively, are required to ensure chemical–mechanical polishing stability [38]. The measured hardness and elastic modulus met this requirement, which allowed for IMD application.

**Figure 9:**
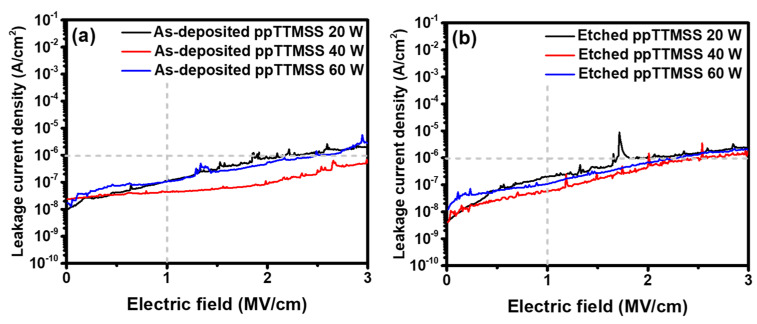
Leakage-current densities of (**a**) as-deposited and (**b**) etched ppTTMSS films as a function of deposition plasma power.

Figure 9a,b show the leakage-current densities of the as-deposited and etched ppTTMSS films, respectively. The leakage-current densities for the as-deposited and etched ppTTMSS films were lower than 10^−6^ A/cm^2^ at 1 MV/cm, which is generally required for the IMD material [17]. At the electric field below 3 MV/cm, a breakdown was not observed for any of the ppTTMSS films. The ppTTMSS films in this study are potentially suitable IMD materials because of their sufficient electrical insulating properties. 

According to the technical roadmap for semiconductor devices and systems, good mechanical strength of low-*k* materials along with a reduction in the *k*-value are the key factors in the interconnects with the continuous scaling down [39]. The ppTTMSS films used in this study showed good mechanical strength to resist the etching process and to meet the chemical–mechanical polishing stability and maintained their *k*-values below 4.0 and their leakage-current densities lower than 10^−6^ A/cm^2^ at 1 MV/cm after the etching process; thus, they can be applied as IMD materials to solve various problems in the scale-down challenge.

## 4. Conclusions

In this study, ppTTMSS films were deposited by PECVD with deposition plasma powers ranging from 20 to 60 W. Subsequently, the ppTTMSS films were etched using the RIE technique. The *k* values of as-deposited and etched ppTTMSS films ranged from 2.33 to 3.76 and from 2.31 to 3.65, respectively. The *n* values of the as-deposited ppTTMSS films were in the range of 1.46 to 1.60, similar to the *n* values of the etched ppTTMSS films (1.45 to 1.58). As the deposition plasma power increased, the density of the ppTTMSS films increased due to ion bombardment. As a result, the hardness and elastic modulus of the ppTTMSS films increased from 1.06 and 6.16 GPa to 8.56 and 52.45 GPa as the deposition plasma power increased. In the FTIR spectra of the ppTTMSS films after RIE etching, there were no significant changes in the peak-area ratios of Si-(CH_3_)_x_ (x = 1, 2, 3), Si-O-Si, Si-CH_3_, and C-H_x_ (x = 1, 2). In contrast, the effects of RIE etching on the surfaces of ppTTMSS films were analyzed by XPS. The oxygen concentration increased but the carbon concentration decreased after RIE etching because of the reaction between the CF_4_/O_2_ plasma and the film surface. The refractive index (*n*) and dielectric constant (*k*) remained almost the same after RIE etching. The leakage-current densities of the as-deposited and etched ppTTMSS films were measured below 10^−6^ A/cm^2^ at 1 MV/cm, which generally meets the requirements for IMD materials.

## Figures and Tables

**Table 1:** Peak-area ratios of deconvoluted Si-O-Si peaks as suboxide, network, and cage structures.

Sample	Deposition Plasma Power (W)	Deconvoluted Peak-Area Ratio of Si-O-Si Peak (%)		
		Suboxide	Network	Cage
As-deposited ppTTMSS	20	27	45	28
	40	45	31	23
	60	56	22	22
Etched ppTTMSS	20	27	45	28
	40	45	31	23
	60	56	22	22

**Table 2:** XPS Atomic concentration of the as-deposition and etched ppTTMSS films.

Sample	Deposition Plasma Power (W)	XPS Atomic Concentration (%)			
		Si	C	O	F
As-deposited ppTTMSS	20	28.11	43.52	28.37	-
	40	27.71	42.92	29.37	-
	60	26.56	42.48	30.96	-
Etched ppTTMSS	20	29.17	14.92	53.67	2.24
	40	28.08	20.00	49.76	2.13
	60	24.74	31.00	42.24	1.99

**Table 3:** Peak-area ratios of the deconvoluted C1s peak as C-Si, C-H/C-C, and C-O C-CF peaks.

Sample	Deposition Plasma Power (W)	Deconvoluted Peak-Area Ratio of C1s Peak (%)			
		C-Si (283.7 eV)	C-H/C-C (284.7 eV)	C-O (286.3 eV)	C-CF (288 eV)
As-deposited ppTTMSS	20	12	80	8	-
	40	12	79	9	-
	60	12	80	8	-
Etched ppTTMSS	20	14	45	33	8
	40	13	56	28	3
	60	13	69	16	2

## Data Availability

The data can be provided by the authors upon request.
